# Accounting for cellular heterogeneity is critical in epigenome-wide association studies

**DOI:** 10.1186/gb-2014-15-2-r31

**Published:** 2014-02-04

**Authors:** Andrew E Jaffe, Rafael A Irizarry

**Affiliations:** 1 Lieber Institute for Brain Development, Johns Hopkins Medical Campus and Department of Biostatistics, Johns Hopkins Bloomberg School of Public Health, Baltimore, MD 21205, USA; 2 Biostatistics and Computational Biology, Dana Farber Cancer Institute and Department of Biostatistics, Harvard School of Public Health, Boston, MA 02115, USA

## Abstract

**Background:**

Epigenome-wide association studies of human disease and other quantitative traits are becoming increasingly common. A series of papers reporting age-related changes in DNA methylation profiles in peripheral blood have already been published. However, blood is a heterogeneous collection of different cell types, each with a very different DNA methylation profile.

**Results:**

Using a statistical method that permits estimating the relative proportion of cell types from DNA methylation profiles, we examine data from five previously published studies, and find strong evidence of cell composition change across age in blood. We also demonstrate that, in these studies, cellular composition explains much of the observed variability in DNA methylation. Furthermore, we find high levels of confounding between age-related variability and cellular composition at the CpG level.

**Conclusions:**

Our findings underscore the importance of considering cell composition variability in epigenetic studies based on whole blood and other heterogeneous tissue sources. We also provide software for estimating and exploring this composition confounding for the Illumina 450k microarray.

## Background

Epigenome-wide association studies (EWAS) of human disease are becoming increasingly common. DNA methylation (DNAm) is of particular interest because it is dynamic across the lifetime, affected by environmental insults, and previously implicated in developmental disorders and cancer [1]. In these studies, DNAm levels are measured genome-wide at thousands to millions of sites in hundreds of individuals to identify loci where these levels are associated with quantitative traits or disease [1,2]. Because existing cohort studies that extensively characterize participants often store blood samples, the most widely available tissue for subsequent/retrospective EWAS is whole blood. Furthermore, many studies measure genome-wide DNAm in blood as obtaining disease-relevant tissues is often invasive and/or impossible. With many of these studies completed, few disease-associated loci have been reported outside of cancer [3], type 1 diabetes [4], and rheumatoid arthritis [5]. Instead a series of papers reporting age-related changes of DNAm profiles have been published [6-14].

Age-related changes in DNAm have been previously reported and functionally described by Chu *et al*. [15]. In this carefully designed study, fluorescence-activated cell sorting (FACS) was used to separate peripheral blood into pure cellular populations. DNAm was measured in four genomic regions, selected using biological insight, and modest age-related changes were found in CD4+ and CD8+ T cells. In contrast, the above-mentioned EWAS measured DNAm for all CpGs selected by the array manufacturers and used whole blood as a source tissue. Whole blood is a heterogeneous collection of different cell types, each with a very different DNA methylation profile [16,17]. Observed whole blood DNAm profiles are therefore mixtures of the cell type profiles. In a seminal paper, Houseman *et al*. [16] describe a statistical method that can accurately estimate relative proportions of cell type components in whole blood. Using practically the same statistical approach, Guintivano *et al*. [18] describe a method for estimating neuron and non-neuron components in brain samples. However, currently there are no published statistical solutions to parsing age effects by cell type from observed whole blood DNAm measurements.

We examined data from five publicly available studies (Additional file 1) and found strong evidence of cell composition changes across age. Furthermore, we find high levels of confounding between age-related variability and cell composition. We report findings that underscore the importance of accounting for cell composition variability in epigenetic studies based on whole blood and other heterogeneous tissue sources.

## Results and discussion

### DNAm profiles show large between cell type differences

We downloaded Illumina HumanMethylation450 BeadChip (Illumina 450k) data from flow-sorted neutrophils (granulocytes), lymphocytes (CD8+ and CD4+ T cells, CD56+ natural killer cells and CD19+ B cells) and CD14+ monocytes from six adult male samples (mean age 38 ± 13.6 years) as previously described [17] and confirmed that sorted blood cell types have unique DNAm profiles (Figure S1 in Additional file 2). In fact 63.5% of the CpGs on the Illumina 450k array showed differences with *P* < 0.05 across these cell types (Figure S1C in Additional file 2).

**Figure 1:**
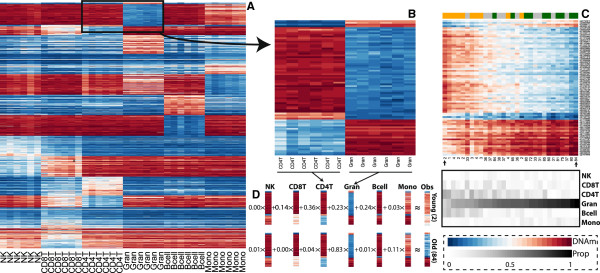
**Illustration of how blood composition drives observed age differences. (A)** Heatmap of the cell sorted data shows very clear and consistent DNAm profiles for each cell type. We show 600 probes selected for estimating composition proportions used to demonstrate differences here. **(B)** To simplify the illustration we selected a section of **(A)** displaying only the two most abundant cell types: CD4+ T cells and granulocytes. **(C)** Heatmap of a randomly selected sample of 30 whole blood samples (from the data in Additional file 1) across three age groups (10 per group): between 1 and 5 years of age, between 30 and 40, greater than 60 years. The same probes as in **(B)** are used. When the samples are ordered by their estimated granulocyte proportion, the samples roughly cluster by age and a similar pattern to **(B)** is observed. The estimated cell count proportions for each of the samples are shown below. Note the strong confounding between age and cell composition. **(D)** For the two samples highlighted with an arrow in **(C)**, we show how a weighted average of the cell type profiles can reconstruct the observed DNAm profiles. The numbers shown are the estimated proportions. Note how different weights (cell counts) for old and young result in very different observed DNAm patterns. Note that the differences in CD4+ T cells and granulocytes drive much of the differences in DNAm. NK, CD56+ natural killer cells; CD8T, CD8+ T cells; CD4T, CD4+ T cells, Gran, granulocytes; Bcell, CD19+ B cells; Mono, CD14+ monocytes; DNAm, proportion of DNA methylation at individual CpGs (Illumina 'beta' values, bound between 0 and 1); Prop, cell count proportion, between 0 and 1 for each component, such that they sum to 1.

We used these data to adapt the statistical method developed by Houseman *et al.*[16] for the Illumina HumanMethylation27 BeadChip (Illumina 27k) array to estimate cell composition from DNAm profiles obtained with its successor, the Illumina 450k. We select a subset of 600 cell-type-specific CpGs (Figure 1) and then use these to estimate proportions in whole blood samples (see Materials and methods). We provide a table with statistical summaries of cell-type variability for all CpGs on the Illumina 450k array (Additional file 3).

### In sorted samples, cell type explains a larger percentage of variability than age

Given these results, for the purposes of our analysis, we assumed that, for the selected 600 CpGs, the cell type-specific DNAm profiles are the same for all ages. Although we know this assumption does not hold true for all CpGs [15], the results of this section suggest that it is reasonable for most CpGs, and our 600 CpG profile in particular. To demonstrate this, we interrogated two publicly available datasets - the Reinius *et al*. [17] Illumina 450k data on 6 men (sample ages were obtained from the authors) and Illumina 27k data from sorted CD4+ T cells and monocytes [6] on 24 and 26 subjects, respectively (see Materials and methods). First, we removed CpG probes that showed age associations (at *P* < 0.05) in the Reinius *et al*. [17] dataset when picking cell-type-discriminating probes for the cell composition estimation. Additionally, in Rakyan *et al*. [6] (which was a larger sample) we found that the percentage of variance explained by cell type was much greater than that explained by age within each cell type with most CpGs showing no significant association with age (Figure S2 in Additional file 2). Furthermore, among the 23 CpGs appearing on the Illumina 27k array that were among the 600 cell-type discriminating CpGs (from the Illumina 450k), only one probe (cg03439703) had a p-value < 0.05 when testing for association with age in both CD4+ T cells (*P* = 0.003) and monocytes (*P* = 0.047).

### Varying cell composition may explain apparent age-associated differences

We downloaded all publicly available DNAm studies in peripheral blood measured with the Illumina 450k array (Additional file 1), re-normalized the data, and applied our method to obtain cell composition estimates for each sample. Note that only three of the studies were focused on finding age-related changes in DNAm [8,10,11], but all studies recorded age information. Figure 1 demonstrates that peripheral blood samples indeed appear to be a mixture of pure cellular components, and differences in DNAm may potentially arise merely from differences in the relative proportions of these components rather than site-specific changes in specific cellular populations (Figure 1C).

### Cell type proportions change with age following monotonic patterns

**Figure 2:**
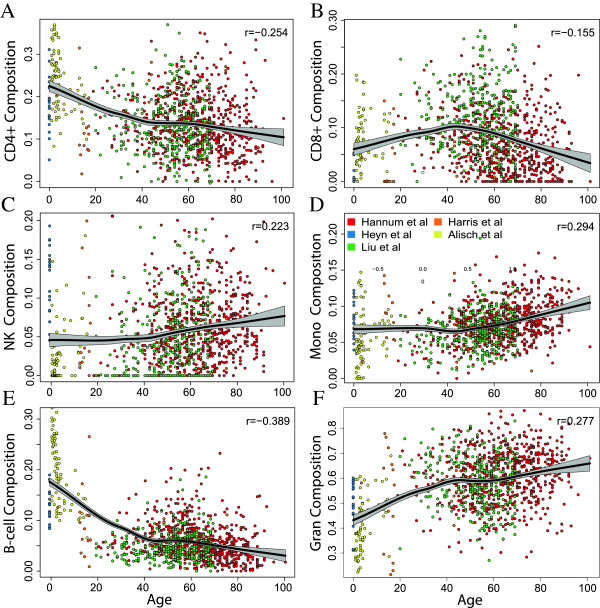
**Cellular composition changes across the lifespan.** Estimated cellular composition proportions are plotted against age for **(A)** CD4+ T cells, **(B)** CD8+ T cells, **(C)** natural killer (NK) cells, **(D)** monocytes (Mono), **(E)** B cells, and **(F)** granulocytes (Gran). Color indicates the data source, which are described in Additional file 1. The black lines are curves fit to data with local weighted regression (loess) with confidence intervals in grey. Spearman correlation coefficients are reported for each composition proportion estimate and age.

We observed consistent age-related changes for the proportions of each cell type (Figure 2). These results are in line with previously published findings related to T cells, namely the involution of the thymus, where T cells in lymphocytes mature. This process begins very early in life [19] and continues with age - the size of the thymus drops approximately 3% per year until the mid-60s, and is approximately 5% the size of the thymus in a newborn [20], suggesting that the number, and diversity, of T cells decreases with age. However, we also note these age-cell count relationships, although monotonic, were non-linear with an inflection point around 40 years (Figure 2). While these findings may be partially attributable to 'batch' effects (given the strong correlation between age and study/dataset), datasets with overlapping age ranges (Liu *et al.* and Hannum *et al.*) have consistent age composition trends (Figure S3 in Additional file 2).

### Cellular composition correlates strongly with global DNAm profiles

**Figure 3:**
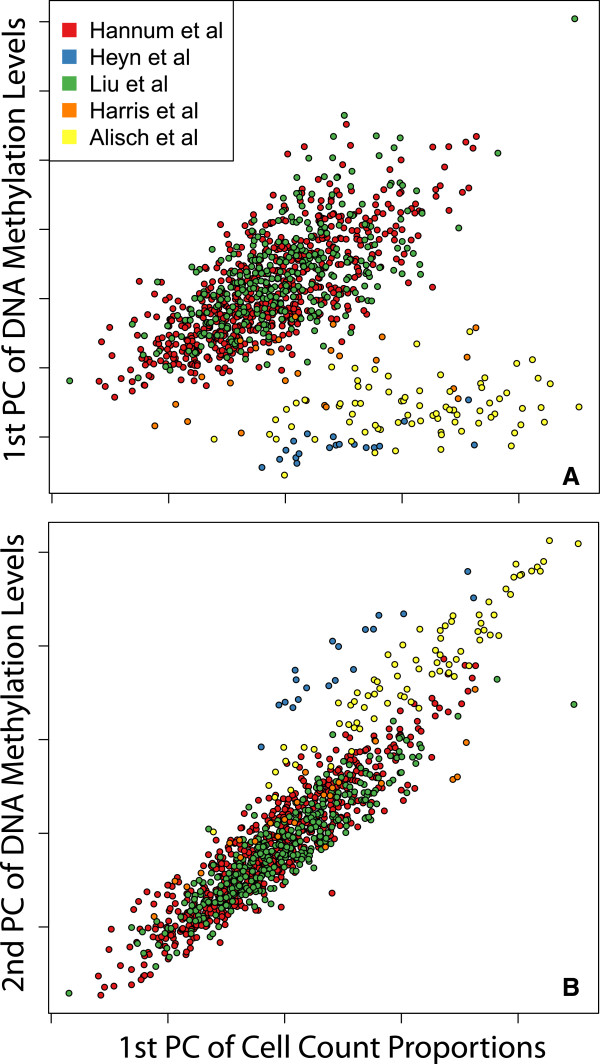
**Cellular composition is a major source of variability in DNAm datasets in whole blood.** Principal components (PCs) **(A)** 1 and **(B)** 2 of the 456,655 DNAm probes (y-axis) and the first PC of the empirical cell counts (x-axis) are highly correlated. The first PC of the DNAm data explains 10.9% of the variance, and the second explains 9.3% of the variance. Color indicates data source, which are described in Additional file 1.

Given that blood cell types have very different DNAm profiles (Figure S1 in Additional file 2) and that cell type proportions change across age (Figure 2), we assessed if cell composition was a major source of variability in the five peripheral blood data sets. We computed the first two principal components of the epigenome-wide DNAm profiles across the five studies and compared them to the first principal component of the cell proportion estimates (Figure 3). The correlation between DNAm variance and composition variance was apparent within each study, often to a stronger degree (Figure S4 in Additional file 2). These observed correlations therefore empirically demonstrate that cell composition is a very large source of variability in DNAm data derived from peripheral blood.

### Confounding between cell composition and age leads to false positives

To determine the adverse effects at the single locus level of the observed confounding between age, cell composition, and DNAm, we reexamined the CpGs reported in the literature to be associated with age [6-13] across several different measurement platforms (Additional file 4). For each of the CpGs reported to associate with age on the Illumina 450k array (n = 134,489), we tested between-to-within cell type variability on the sorted DNAm data and found that 86.7% of these had *P* < 0.05 across cell type (Figure S5 in Additional file 2).

**Figure 4:**
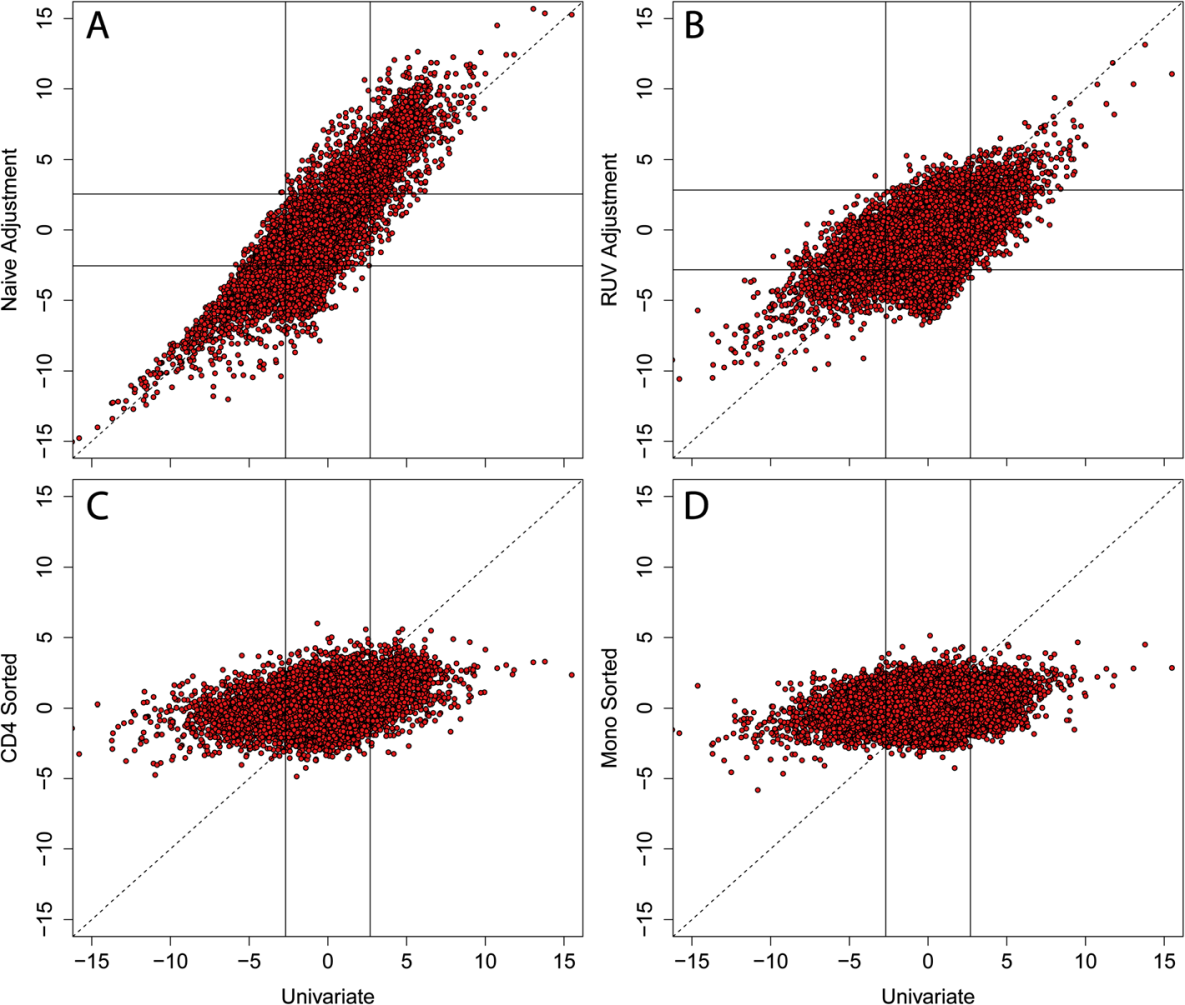
**Confounding between cellular composition and age at the CpG level.** Comparisons between resulting t-statistics for age on DNA methylation levels in Hannum *et al*. [10] using **(A)** naïve (for example, including cell composition estimates as covariates in regression models), **(B)** two-step Remove Unwanted Variation (RUV), **(C)** flow-sorted CD4+ T cells and **(D)** flow-sorted monocytes compared to the effect of age on DNAm in a univariate model. The univariate and naïve models also adjusted for processing plate, which was a very strong confounder. Here, analysis with RUV attenuates the association between DNAm and age. The solid lines indicate the resulting t-statistic cutoff for false discovery rate <5% - no probes were significant at this threshold in the cell sorted data. All panels contain probes present on both the Illumina 450k and 27k (n = 24,692) to facilitate comparisons to age associations in the flow-sorted cellular populations.

A simple linear regression model including the cell composition percentages as covariates has been suggested as a way to adjust for the confounding [5]. We applied this method to the data from Hannum *et al*. [10] and Alisch *et al*. [8] and found that the adjusted estimates are, on average, closer to 0 (Figure S6A in Additional file 2). However, at this level of confounding it is not clear that this naïve approach will in fact produce unbiased adjusted estimates (Figure 4A) [21]. We therefore tried two alternative approaches. First we applied the Remove Unwanted Variation (RUV) method [22], an analysis that estimates and adjusts for unknown surrogate variables as done by Leek and Storey [23]. This resulted in much greater, but not complete, attenuation of the age association estimates (Figure 4B; Figure S6B in Additional file 2). Next we obtained age association estimates from fitting the model to data from sorted CD4+ T cells and granulocytes. Note that in these data, cell composition is not a confounder and we see minimal evidence of age association (Figure 4C,D; Figure S6C,D in Additional file 2). We did not implement the adjustment approach suggested by Guintivano *et al*. [18] because mathematical derivations demonstrated their solution adjusts for confounding in special situations (see Materials and methods).

### Improved biological interpretation after composition filtering

We removed results from Johansson *et al*. [14] (which reporting one-third of the array was differentially methylated) then mapped the remaining 5,237 age-associated CpGs (Additional file 4) to human genes using the database provided by Triche [24]. For each Gene Ontology category [25] with more than 25 annotated gene IDs we counted the number of CpGs associated with a gene in that category and formed an observed count to expected count ratio (see Materials and methods). We then filtered this list by removing CpGs associated with cell composition and recomputed the observed to expected ratios. With the unfiltered list, 10 of the top 20 enriched categories were clearly related to the immune system while only three were related to development, whereas in the filtered list 9 of the top 20 were associated with developmental processes and only 4 to immune response (Additional file 5).

## Conclusions

Whole blood has been one of the most widely used source tissues in EWAS. Here we demonstrate that, in these studies, cellular composition explains much of the observed variability in DNAm. Therefore, when the outcome of interest correlates with cell composition, as age does, failure to account for cellular heterogeneity may result in many false positives. For binary outcomes, for example, we may observe differences between cases and controls, not due to the real differences in DNAm, but rather due to cases and controls having different blood cell counts (Figure 1).

While our re-analysis of publicly available data does not necessarily suggest that all reported age-related DNAm changes in blood are false positives, it certainly suggests that one should account for cellular composition. We therefore recommend that users of the Illumina 450k array studying whole blood perform the cell composition estimation (using, for example, the *estimateCellCounts* function we have added to the *minfi* Bioconductor package) and check for possible confounding. If confounding is present, we recommend the use of our table (Additional file 3; also available in the *FlowSorted.Blood.450k* Bioconductor package) that summarizes cell-type variability for each CpG. Those CpGs with methylation values highly associated with cell-type variability should be treated with skepticism, and we strongly recommend that CpGs associated with both composition and the covariate of interest be validated using FACS-derived cellular populations.

Note that due to the high levels of confounding we currently do not recommend regression approaches for adjustment purposes, but we note that RUV performed best for reducing the composition-based confounding. However, when there is no or minimal confounding, the added unaccounted variability may result in false negatives. In such cases popular factor-based 'batch' correction methodology, like surrogate variable analysis [23], and RUV [22] can empirically estimate and control for cell-type composition.

Note that these confounding problems are not confined to blood, but rather any tissue source that contains a mixture of cell types. Here, careful study design, via targeted validation employing cell sorting within the tissue of interest, can help isolate cell type-specific changes, such as age-related DNAm changes in the pure cellular populations of blood beyond the preliminary negative findings in CD4+ T cells and monocytes from Rakyan *et al*. [6]. These may better explain observed biological effects, specifically, which epigenetic marks mediate risk for disease or associate with a trait. Characterizing and exploring the effects of cellular heterogeneity is therefore a necessary step in the analysis of genome-wide DNAm data in any heterogeneous tissue source, especially peripheral blood.

## Materials and methods

### Sample and study selection

There were five publicly available datasets on the Illumina 450k platform [5,8,10,11,26] performed on blood samples in the Gene Expression Omnibus (GEO) available through the National Center for Biotechnology Information (NCBI) as of February 2013 [27]. We also downloaded cell sorted data described in the Results section from Reinius *et al*. [17] (GSE35069). Because study and age were almost perfectly confounded, and because there were very strong effects of study in the processed GEO data, we required 'raw' methylated (M) and unmethylated (U) channels from the Illumina 450k to preprocess and normalize all of the samples together, including the cell-sorted dataset. One study, Horvath *et al.*[12], was not included in the manuscript because the GEO entry lacked raw data. Samples were dropped according to three criteria: 1) missing an age in the database (N = 11); 2) known to be cell-sorted, according to published manuscripts (N = 2, from Heyn *et al.*[11]); and 3) hypothesized to be cell-sorted, based on granulocyte count values (Figure S7 in Additional file 2), including all centenarian samples from Heyn *et al* (N = 19), as all appeared to be only granulocytes, and 21 samples from Harris *et al.*[26], which appeared to be granulocyte-depleted (the manuscript refers to a subset of samples being sorted, but it was not available information in the GEO entry). This left 1,098 samples across 5 studies.

We performed across-array quantile normalization within the M and U channels separately to normalize intensities across samples. Before normalization, we dropped probes on the sex chromosomes (chromosome X = 11,232 and chromosome Y = 416) and also probes that contained an annotated SNP (via dbSNP 137 Common database) in the CpG site (N = 16,756) and at the single base extension site (N = 7,880). This left 456,655 autosomal probes across the epigenome. After normalization, DNAm measurements on the logit scale were calculated as log_2_(M/U), and then transformed to Illumina’s 'beta' scale (proportion methylation, between 0 and 1). This approach is similar to the 'ABNorm' approach described by Sun *et al*. [28], but we use the logit transform described above rather than the Illumina approach [M/(M + U + 100)] for calculating the beta values.

### Empirically estimating cellular composition using the Illumina 450k microarray

We tailored the algorithm designed by Houseman *et al*. [16] for the Illumina 27k array to the Illumina 450k array. Briefly, the Houseman algorithm identified 500 CpGs that discriminated cellular composition in flow-sorted cell populations (consisting of CD4+ and CD8+ T cells, B cells, monocytes, natural killer cells, and granulocytes). The algorithm then fits a nonlinear random effects model at each of these CpGs, estimating the coefficient for each cellular component, and then uses these coefficients to predict the relative proportion of each cellular component in peripheral blood samples.

However, there were several reasons that prevented the direct use of Houseman *et al*.’s algorithm on the 1,098 blood samples obtained on the Illumina 450k. First, while 473 of the 500 composition-discriminating CpGs were present on the Illumina 450k, these probes exhibited slightly different behavior in the two arrays (Figure S8 in Additional file 2). Second, 291 CpGs used by Houseman *et al*.’s algorithm contained an annotated SNPs (by rs number in the dbSNP137 database) at the CpG site of interest (N = 57), at the single base extension site following the CpG (N = 34) or in the probe sequence itself (N = 200). Problems detailing the inclusion of SNPs in the design of the Illumina 450k have been discussed previously [29,30], and given our data are from a genetically heterogeneous population, we elected to exclude some of these probes.

We therefore obtained flow-sorted data, including the same six cellular components on six adult male subjects on the Illumina 450k platform [17], and derived our own similar blood composition algorithm using linear modeling across 600 composition-discriminating probes. We computed t-statistics for each cell type after removing probes that associated with age (at *P* ≤ 0.05), comparing that particular cell type with all others, and selected among the CpGs showing differences at *P* < 10^-8^ the 100 most differentially methylated probes by effect size, 50 hypermethylated and 50 hypomethylated. One outlying CD8+ T cell was excluded for the sake of composition estimation. The choices of 50 and 10^-8^ were somewhat arbitrary, but in-sample cross-validation (via leaving out one sample per cell type, training the model on the remaining 30 samples, and then predicting the 6 left out samples) demonstrated nearly perfect concordance between our estimates of cellular composition and the true values (Figure S9 in Additional file 2).

We also validated the overall algorithm using publicly available brain data from Guintivano *et al*. [18], which consisted of flow-sorted NeuN + and NeuN- cellular populations from the dorsolateral prefrontal cortex as training data, and then mixture data containing 10% NeuN+/90% NeuN-, 20% NeuN+/80% NeuN-, …, 90% NeuN+/10% NeuN- and bulk tissue data with FACS-derived counts of NeuN + cells as testing data. We processed the data (quantile normalization, dropping probes with SNPs and on sex chromosomes), picked 50 hypo- and hyper-methylated probes, and implemented the algorithm. The algorithm successfully recovered the mixture experiment (correlation = 0.9995; Figure S10A in Additional file 2) and predicted the FACS-derived counts from bulk tissue with moderate accuracy (correlation = 0.786). Lastly, we expect similar accuracy in blood (<10% on the Illumina 27k) as Houseman *et al*. [16], as we have adapted the algorithm to the Illumina 450k without changing the regression calibration approach.

Software to implement the estimation of cellular compositions from cell-sorted DNAm data is available in the *minfi* Bioconductor package [31]. Publicly available cell-sorted data, to be used in conjunction with the *minfi* package, are available in the *FlowSorted.Blood.450k* Bioconductor package.

### Previously published solution does not generally adjust for confounding

Guintivano *et al*. [18] also provide software that implements a method that they claim can transform data to eliminate (or at least reduce) the confounding effect of cell type heterogeneity on methylation profiles. Although the software is developed for brain, and only for two cell types, one could envision extensions applicable to cases with more cell types such as blood.

However, we offer a mathematical proof demonstrating that the solution offered in the paper only adjusts for confounding in a very special case. To understand the transformation we downloaded the accompanying software package CETS (version 0.99.2) and deciphered it from the R code. Here is a mathematical description of what the transformation does.

Let *Y*_*i*_ be the observed methylation profile for the *i*th individual, a mix of glia (G) and neurons (N). Then we can write:

$$Y_{i}=\pi_{i}{\mu_{i}}_{,N}+\left(1-\pi_{i}\right){\mu_{i}}_{,G}+\epsilon_{i}$$

where *π*_*i*_ is the proportion of the *i*th sample that comes from neurons, *μ*_*i,N*_ and *μ*_*i,G*_ are the profiles for neurons and glia, respectively, and *ϵ*_*i*_ is measurement error. In their software, Guintivano *et al*. [18] provide neuron and glia profiles based on an average across many cell-sorted samples, which we will denote with ${\bar{\mu }}_{N}$ and ${\bar{\mu }}_{G}$. It is important to note that these are averages and thus different from the individual profiles. The transformation proposed by Guintivano *et al*. [18] is:

$$T\left(Y_{i}\right)=Y_{i}+\left(1-\pi_{i}\right)\left({\bar{\mu }}_{N}-{\bar{\mu }}_{G}\right)$$

They claim that this will recover the pure neuronal signal *μ*_*i,N*_*.* But we can do some arithmetic to note that the above can be rewritten as:

$$\mu_{i,N}+\left(1-\pi_{i}\right)\left[\left({\bar{\mu }}_{N}-\mu_{i,N}\right)-\left({\bar{\mu }}_{G}-\mu_{i,G}\right)\right]+\epsilon_{i}$$

Thus, the signal is recovered only when the difference between the individual profiles and the average profiles are the same across cell type, which is not a reasonable, nor useful, assumption.

### Variability in sorted cell populations

We downloaded publicly available data from Rakyan *et al*. [6] at GEO accession GSE20242, which consisted of sorted adult blood samples for monocyte and CD4+ T-cell populations. Linear regression models including i) age, ii) cell type, iii) both age, cell type, and their interaction term were fit at every probe. We summarized each fit with the adjusted R^2^ (coefficient of determination) . We then examined the *P*-values for the age terms within each cellular population at our 600 probes from the Illumina 450k used to estimate cellular composition that were also present on the Illumina 27k (n = 23).

### Analysis of reported age-associated differentially methylated regions

We downloaded tables for statistically significant age-associated differentially methylated probes or regions (DMRs) from the supplementary material of published manuscripts listed in Additional file 4. For each reported age-associated DMR, we identified the F-statistic (and resulting marginal *P*-value) for that probe for the effect of composition in the publicly available sorted Illumina 450k data [17].

We applied naïve regression adjustment (for example, adjusting for cell type estimates) and two-step RUV using k = 10 (principal components) in Alisch *et al*. [8] and k = 30 in Hannum *et al*. [10], which were determined using diagnostic plots across a range of k values. Univariate regression modeling for Hannum *et al*. [10] included a categorical 'plate' adjustment variable, as plate and age were strongly associated, and plate and DNAm estimates were also associated. The RUV method requires control probes that are affected by the confounder (cell composition) but not the outcome of interest. We therefore used our 600 probes used to estimate cell type proportion since we showed these had no relation to age in at least two cell types. While it is possible that they are age associated in other cell types the results summarized in Figure S2 in Additional file 2 suggest that this is a useful approximation. With these control probes in place we then let the algorithm estimate the surrogate variables.

We assessed functional significance through enrichment using pre-defined gene sets with the Gene Ontology database. First we mapped each CpG to its Entrez Gene ID [24] for background enrichment (311,817/456,655 probes had an annotated Entrez Gene ID). For each gene set with 25 or more genes, we assessed the number of CpGs that mapped to each gene set. Then we assessed the number of reported age DMR CpGs in the existing gene sets, before (n = 4,691/5,237 mapped to an Entrez ID) and after (n = 1,090/1,209 mapped to an Entrez ID) removing probes that correlated with composition (f-statistic *P*-value <1 × 10^-4^ and DNAm range >10%). The observed verses expected ratios were computed for every gene set before and after this composition filtering, and are presented in Additional file 5.

### Data availability

All datasets are publicly available in the GEO database [27] at the accessions available in Additional file 1.

## Abbreviations

DNAm: DNA methylation; EWAS: epigenome-wide association study; FACS: fluorescence-activated cell sorting; GEO: Gene Expression Omnibus; M: methylated; RUV: Remove Unwanted Variation; SNP: single-nucleotide polymorphism; U: unmethylated.

## Competing interests

The authors declare that they have no competing interests.

## Authors’ contributions

AEJ and RAI conceived the study, performed data analysis, and wrote the manuscript. AEJ implemented the analysis and wrote the code. Both authors read and approved the final manuscript.

## Supplementary Material

Additional file 1: Table S1 Studies included in the cellular composition analyses. 'Dataset' refers to each study used in the paper, followed by its citation (see References for full citation); 'N' is the number of samples included from each study; 'GEO ID' is the Gene Expression Omnibus identifier; 'Primary Outcome' is the main disease or trait reported by the referenced article - note that only some datasets were primarily focused on age; 'Median Age [IQR] (yrs)' is the median age of the study participants, followed by their interquartile range (25^th^ percentile, 75^th^ percentile), in years. Click here for file

Additional file 2: Figure S1 Differential DNA methylation by cell composition. **Figure S2.** Contributions of age and cell type to cell-sorted DNAm data. **Figure S3.** Age versus cell type for Liu *et al.*[5] and Hannum *et al.*[10] studies. **Figure S4.** Global variation in DNA methylation by composition, by study sample (Additional file 1). **Figure S5.** Composition *P*-values from previously reported age-associated differentially methylated regions. **Figure S6.** Composition confounding in Alisch *et al*. [8]. **Figure S7.** Removal of samples with outlying granulocyte counts. **Figure S8.** Differences between sorted profiles on the Illumina 27k versus the Illumina 450k. **Figure S9.** Cross-validated cell counts. **Figure S10.** Validation of algorithm using brain data. Click here for file

Additional file 3: Table S2 Association of each probe on the Illumina 450k with blood cell composition. Note that probes on the sex chromosomes and those that contain annotated SNPs have been filtered (see Materials and methods). We recommend using the CpG identifiers to match each probe from a user’s differential methylation analysis in their whole blood data to obtain the corresponding composition *P*-value - if there are many small *P*-values for significant differentially methylated sites for the exposure/outcome/trait of interest, this may be a sign of confounding via composition differences, in which case we recommend estimating cellular components using the *minfi* Bioconductor package, and formally exploring this potential correlation between the trait, composition, and DNAm. 'Name' refers to the CpG identifier from the Illumina 450k; 'Fstat' and 'p.value' are the f-statistic and corresponding *P*-value for composition from the ANOVA containing six samples/biological replicates per cell type across six cell types (n = 36; see Materials and methods); 'CD8T_mean' is the mean DNAm across the six CD8+ T cell replicates, on the beta/proportion methylation scale; 'CD4T_mean' is the mean DNAm across the six CD4+ T-cell replicates, on the beta/proportion methylation scale; 'NK_mean' is the mean DNAm across the six natural killer cell replicates, on the beta/proportion methylation scale; 'Bcell_mean' is the mean DNAm across the six B-cell replicates, on the beta/proportion methylation scale; 'Mono_mean' is the mean DNAm across the six monocyte replicates, on the beta/proportion methylation scale; 'Gran_mean' is the mean DNAm across the six granulocyte replicates, on the beta/proportion methylation scale; 'DNAm_min' and 'DNAm_max' are the minimum and maximum beta values, respectively, across the 36 samples at each loci; 'DNAm_range' is the range of beta values. Click here for file

Additional file 4: Table S3 Previously published results for age-associated differential methylation in blood. 'Study (Reference)' refers to a particular study, along with its reference, that reported age-associated differentially methylated regions (aDMRs); 'Platform' is the DNA methylation microarray platform used by the study - '450k' is the Illumina 450k, '27k' is the Illumina 27k and 'CHARM 2.0' is the second generation of the Comprehensive High-Throughput Arrays for Relative Methylation platform. '# of aDMRs' reports the number of differentially methylated loci associated with age - the number left of the backslash is the number reported at genome-wide significance (determined by respective publication) and to the right, the number of significant sites available as a Supplementary Table obtained from each respective manuscript; 'SVA?' displays whether surrogate variable analysis was used in the paper, which may have partially adjusted for blood cell composition effects. Click here for file

Additional file 5: Table S4 Gene Ontology (GO) enrichment before and after removing Illumina 450k probes associated with cellular composition. 'GO ID' refers to the GO identifier; 'Background' refers to all of the probes on the Illumina 450k that mapped to an Entrez Gene ID; 'Before' refers to age-associated probes that were not filtered by whether they associated with cellular composition; 'After' refers to age-associated probes after those probes associated with cellular composition were filtered from the analysis; 'Number of Probes Enriched' is the number of probes that mapped to that GO category for each condition; 'Expected Number of Probes' is the expected number of probes, assuming no enrichment, for each category; 'Observed/Expected Ratio' is the ratio of observed to expected counts, a.k.a. the odds ratio; 'GO Term' is the biological term corresponding to each GO ID; 'Set Size' is the number of genes for each GO set. 'Ontology' refers to the three GO classifications - molecular function ('MF'), biological processes ('BP'), and cellular component ('CC'); 'Rank' refers to the *P*-value rank, smallest to largest, before and after filtering age-associated probes also associated with cellular composition. Click here for file

## References

[B1] Rakyan VK Down TA Balding DJ Beck S Epigenome-wide association studies for common human diseases Nat Rev Genet 2011 12 529 541 21747404 10.1038/nrg3000 PMC3508712

[B2] Jaffe AE Murakami P Lee H Leek JT Fallin MD Feinberg AP Irizarry RA Bump hunting to identify differentially methylated regions in epigenetic epidemiology studies Int J Epidemiol 2012 41 200 209 22422453 10.1093/ije/dyr238 PMC3304533

[B3] Teschendorff AE Menon U Gentry-Maharaj A Ramus SJ Gayther SA Apostolidou S Jones A Lechner M Beck S Jacobs IJ Widschwendter M An epigenetic signature in peripheral blood predicts active ovarian cancer PLoS One 2009 4 e8274 20019873 10.1371/journal.pone.0008274 PMC2793425

[B4] Rakyan VK Beyan H Down TA Hawa MI Maslau S Aden D Daunay A Busato F Mein CA Manfras B Dias KR Bell CG Tost J Boehm BO Beck S Leslie RD Identification of type 1 diabetes-associated DNA methylation variable positions that precede disease diagnosis PLoS Genet 2011 7 e1002300 21980303 10.1371/journal.pgen.1002300 PMC3183089

[B5] Liu Y Aryee MJ Padyukov L Fallin MD Hesselberg E Runarsson A Reinius L Acevedo N Taub M Ronninger M Shchetynsky K Scheynius A Kere J Alfredsson L Klareskog L Ekstrom TJ Feinberg AP Epigenome-wide association data implicate DNA methylation as an intermediary of genetic risk in rheumatoid arthritis Nat Biotechnol 2013 31 142 147 23334450 10.1038/nbt.2487 PMC3598632

[B6] Rakyan VK Down TA Maslau S Andrew T Yang TP Beyan H Whittaker P McCann OT Finer S Valdes AM Leslie RD Deloukas P Spector TD Human aging-associated DNA hypermethylation occurs preferentially at bivalent chromatin domains Genome Res 2010 20 434 439 20219945 10.1101/gr.103101.109 PMC2847746

[B7] Teschendorff AE Menon U Gentry-Maharaj A Ramus SJ Weisenberger DJ Shen H Campan M Noushmehr H Bell CG Maxwell AP Savage DA Mueller-Holzner E Marth C Kocjan G Gayther SA Jones A Beck S Wagner W Laird PW Jacobs IJ Widschwendter M Age-dependent DNA methylation of genes that are suppressed in stem cells is a hallmark of cancer Genome Res 2010 20 440 446 20219944 10.1101/gr.103606.109 PMC2847747

[B8] Alisch RS Barwick BG Chopra P Myrick LK Satten GA Conneely KN Warren ST Age-associated DNA methylation in pediatric populations Genome Res 2012 22 623 632 22300631 10.1101/gr.125187.111 PMC3317145

[B9] Bell JT Tsai PC Yang TP Pidsley R Nisbet J Glass D Mangino M Zhai G Zhang F Valdes A Shin SY Dempster EL Murray RM Grundberg E Hedman AK Nica A Small KS Dermitzakis ET McCarthy MI Mill J Spector TD Deloukas P Epigenome-wide scans identify differentially methylated regions for age and age-related phenotypes in a healthy ageing population PLoS Genet 2012 8 e1002629 22532803 10.1371/journal.pgen.1002629 PMC3330116

[B10] Hannum G Guinney J Zhao L Zhang L Hughes G Sadda S Klotzle B Bibikova M Fan JB Gao Y Deconde R Chen M Rajapakse I Friend S Ideker T Zhang K Genome-wide methylation profiles reveal quantitative views of human aging rates Mol Cell 2013 49 359 367 23177740 10.1016/j.molcel.2012.10.016 PMC3780611

[B11] Heyn H Li N Ferreira HJ Moran S Pisano DG Gomez A Diez J Sanchez-Mut JV Setien F Carmona FJ Puca AA Sayols S Pujana MA Serra-Musach J Iglesias-Platas I Formiga F Fernandez AF Fraga MF Heath SC Valencia A Gut IG Wang J Esteller M Distinct DNA methylomes of newborns and centenarians Proc Natl Acad Sci USA 2012 109 10522 10527 22689993 10.1073/pnas.1120658109 PMC3387108

[B12] Horvath S Zhang Y Langfelder P Kahn RS Boks MP van Eijk K van den Berg LH Ophoff RA Aging effects on DNA methylation modules in human brain and blood tissue Genome Biol 2012 13 R97 23034122 10.1186/gb-2012-13-10-r97 PMC4053733

[B13] Lee H Jaffe AE Feinberg JI Tryggvadottir R Brown S Montano C Aryee MJ Irizarry RA Herbstman J Witter FR Goldman LR Feinberg AP Fallin MD DNA methylation shows genome-wide association of NFIX, RAPGEF2 and MSRB3 with gestational age at birth Int J Epidemiol 2012 41 188 199 22422452 10.1093/ije/dyr237 PMC3304532

[B14] Johansson A Enroth S Gyllensten U Continuous aging of the human DNA methylome throughout the human lifespan PLoS One 2013 8 e67378 23826282 10.1371/journal.pone.0067378 PMC3695075

[B15] Chu M Siegmund KD Hao QL Crooks GM Tavare S Shibata D Inferring relative numbers of human leucocyte genome replications Br J Haematol 2008 141 862 871 18410448 10.1111/j.1365-2141.2008.07142.x

[B16] Houseman EA Accomando WP Koestler DC Christensen BC Marsit CJ Nelson HH Wiencke JK Kelsey KT DNA methylation arrays as surrogate measures of cell mixture distribution BMC Bioinformatics 2012 13 86 22568884 10.1186/1471-2105-13-86 PMC3532182

[B17] Reinius LE Acevedo N Joerink M Pershagen G Dahlen SE Greco D Soderhall C Scheynius A Kere J Differential DNA methylation in purified human blood cells: implications for cell lineage and studies on disease susceptibility PLoS One 2012 7 e41361 22848472 10.1371/journal.pone.0041361 PMC3405143

[B18] Guintivano J Aryee MJ Kaminsky ZA A cell epigenotype specific model for the correction of brain cellular heterogeneity bias and its application to age, brain region and major depression Epigenetics 2013 8 290 302 23426267 10.4161/epi.23924 PMC3669121

[B19] Steinmann GG Klaus B Muller-Hermelink HK The involution of the ageing human thymic epithelium is independent of puberty. A morphometric study Scand J Immunol 1985 22 563 575 4081647 10.1111/j.1365-3083.1985.tb01916.x

[B20] Boyd RL Tucek CL Godfrey DI Izon DJ Wilson TJ Davidson NJ Bean AG Ladyman HM Ritter MA Hugo P The thymic microenvironment Immunol Today 1993 14 445 459 8216723 10.1016/0167-5699(93)90248-J

[B21] Montano CM Irizarry RA Kaufmann WE Talbot K Gur RE Feinberg AP Taub MA Measuring cell-type specific differential methylation in human brain tissue Genome Biol 2013 14 R94 24000956 10.1186/gb-2013-14-8-r94 PMC4054676

[B22] Gagnon-Bartsch JA Speed TP Using control genes to correct for unwanted variation in microarray data Biostatistics 2012 13 539 552 22101192 10.1093/biostatistics/kxr034 PMC3577104

[B23] Leek JT Storey JD Capturing heterogeneity in gene expression studies by surrogate variable analysis PLoS Genet 2007 3 1724 1735 17907809 10.1371/journal.pgen.0030161 PMC1994707

[B24] Triche T Jr IlluminaHumanMethylation450k.db: Illumina Human Methylation 450k annotation data, version 2.0.7 [http://www.bioconductor.org/packages/release/data/annotation/html/IlluminaHumanMethylation450k.db.html]

[B25] Ashburner M Ball CA Blake JA Botstein D Butler H Cherry JM Davis AP Dolinski K Dwight SS Eppig JT Harris MA Hill DP Issel-Tarver L Kasarskis A Lewis S Matese JC Richardson JE Ringwald M Rubin GM Sherlock G Gene ontology: tool for the unification of biology. The Gene Ontology Consortium Nat Genet 2000 25 25 29 10802651 10.1038/75556 PMC3037419

[B26] Harris RA Nagy-Szakal D Pedersen N Opekun A Bronsky J Munkholm P Jespersgaard C Andersen P Melegh B Ferry G Jess T Kellermayer R Genome-wide peripheral blood leukocyte DNA methylation microarrays identified a single association with inflammatory bowel diseases Inflamm Bowel Dis 2012 18 2334 2341 22467598 10.1002/ibd.22956 PMC3812910

[B27] Edgar R Domrachev M Lash AE Gene Expression Omnibus: NCBI gene expression and hybridization array data repository Nucleic Acids Res 2002 30 207 210 11752295 10.1093/nar/30.1.207 PMC99122

[B28] Sun Z Chai HS Wu Y White WM Donkena KV Klein CJ Garovic VD Therneau TM Kocher JP Batch effect correction for genome-wide methylation data with Illumina Infinium platform BMC Med Genomics 2011 4 84 22171553 10.1186/1755-8794-4-84 PMC3265417

[B29] Zhang X Mu W Zhang W On the analysis of the illumina 450k array data: probes ambiguously mapped to the human genome Front Genet 2012 3 73 22586432 10.3389/fgene.2012.00073 PMC3343275

[B30] Beyan H Down TA Ramagopalan SV Uvebrant K Nilsson A Holland ML Gemma C Giovannoni G Boehm BO Ebers GC Lernmark A Cilio CM Leslie RD Rakyan VK Guthrie card methylomics identifies temporally stable epialleles that are present at birth in humans Genome Res 2012 22 2138 2145 22919074 10.1101/gr.134304.111 PMC3483543

[B31] Aryee MJ Jaffe AE Corrada-Bravo H Ladd-Acosta C Feinberg AP Hansen KD Irizarry RA Minfi: A flexible and comprehensive Bioconductor package for the analysis of Infinium DNA Methylation microarrays Bioinformatics 2014 [Epub ahead of print] 10.1093/bioinformatics/btu049 PMC4016708 24478339

