# Supplementary material for: Accounting for cellular heterogeneity is critical in epigenome-wide association studies
Source: Genome Biol. 2014 Feb 4;15(2):R31. doi: 10.1186/gb-2014-15-2-r31 (PMC4053810; doi:10.1186/gb-2014-15-2-r31)
Supplement: Additional file 2: Figure S1 — Differential DNA methylation by cell composition. Figure S2. Contributions of age and cell type to cell-sorted DNAm data. Figure S3. Age versus cell type for Liu et al.[5] and Hannum et al.[10] studies. Figure S4. Global variation in DNA methylation by composition, by study sample (Additional file 1). Figure S5. Composition P-values from previously reported age-associated differentially methylated regions. Figure S6. Composition confounding in Alisch et al. [8]. Figure S7. Removal of samples with outlying granulocyte counts. Figure S8. Differences between sorted profiles on the Illumina 27k versus the Illumina 450k. Figure S9. Cross-validated cell counts. Figure S10. Validation of algorithm using brain data. [file gb-2014-15-2-r31-S2.pdf]

Supplementary Figure 1: Differential DNA methylation by cell composition. (A) Plot of the first two principal components of the cell-sorted Illumina 450k data from Reinius et al. (2012). Color indicates cell type. (B) Heatmap of the 1,000 most variable probes in this dataset, which cluster by cell type. (C) Histogram of p-values from an f-test for composition at each probe on the array. P-values less than $1x10^{-20}$ were thresholded to this value. The dashed vertical line indicates the cutoff of $p = 0.05$.

Supplementary Figure 2: Contributions of age and cell type to cell sorted DNA methylation data. Publicly available Illumina 27k DNAm data from CD4+ T-cells and monocytes was re-analyzed [Rakyan 2010].The adjusted coefficient of determination ("adjusted R^2") was calculated at each probe for models of i) cell type (monocyte vs t-cell) ii) age and iii) the interaction model age by cell type. These model fits demonstrate that, overall, cell type explains a larger proportion of the variance of DNAm than age.

Supplementary Figure 3: Age versus cell type for Liu et al and Hannum et al studies. These two studies had overlapping age ranges, and consistent patterns of composition across age.

Supplemental Figure 4: Global variation in DNA methylation by composition, by study sample (Supplemental Table 1). Y-axis: principal component (PC1) 1 in DNA methylation data and x-axis: PC 1 from cell proportion estimates from six cell types.

Supplementary Figure 5: Composition p-values from previously reported age DMRs. Histogram of composition p-values (from the f-test in Supplementary Figure 1) among the reported age DMRs across 9 studies (in Supplementary Table 3).

Supplementary Figure 6: Composition confounding in Alisch et al (2012). Comparisons between resulting T-statistics for age on DNA methylation levels in Hannum et al. (2012) using (A) naïve (e.g. including cell composition estimates as covariates in regression models), (B) two-step RUV, (C) flow-sorted CD4+ T-cells and (D) flow-sorted monocytes, compared to the effect of age on DNA methylation in a univariate model. Here, analysis with RUV attenuates the association between DNAm and age. The solid lines indicate the resulting t-statistic cutoff for the false discovery rate (FDR) < 5% - there were no probes significant at this threshold in the cell sorted data. All panels contain probes present on both the Illumina 450k and 27k (n=24,692) to facilitate comparisons to age associations in the flow-sorted cellular populations.

Supplementary Figure 7: Removal of samples with outlying granulocyte counts. The estimated granulocyte counts are plotted against age. Boxes indicate samples that were removed from subsequent analyses, as described in the Methods section. Color indicates data source (from Supplementary Table 1).

Supplementary Figure 8: Differences between sorted profiles on the Illumina 27k versus the Illumina 450k. The mean profile across each of 6 cell types and 473 probes, which were the probes used by Houseman et al. (2012) that were also present on the Illumina 450k.

Supplementary Figure 9: Cross-validated cell counts. In each of 6 iterations, one replicate of each cell type was left out, the model was trained on the remaining samples, and the compositions of the left out samples were predicted. The 600 cell type differentiating probes were used in each of the six iterations.

Supplementary Figure 10: validation of algorithm using brain data. Predicting the relative proportion of NeuN+ cells in A) mixtures at various concentrations and B) bulk brain tissue with known FACS-derived proportions.

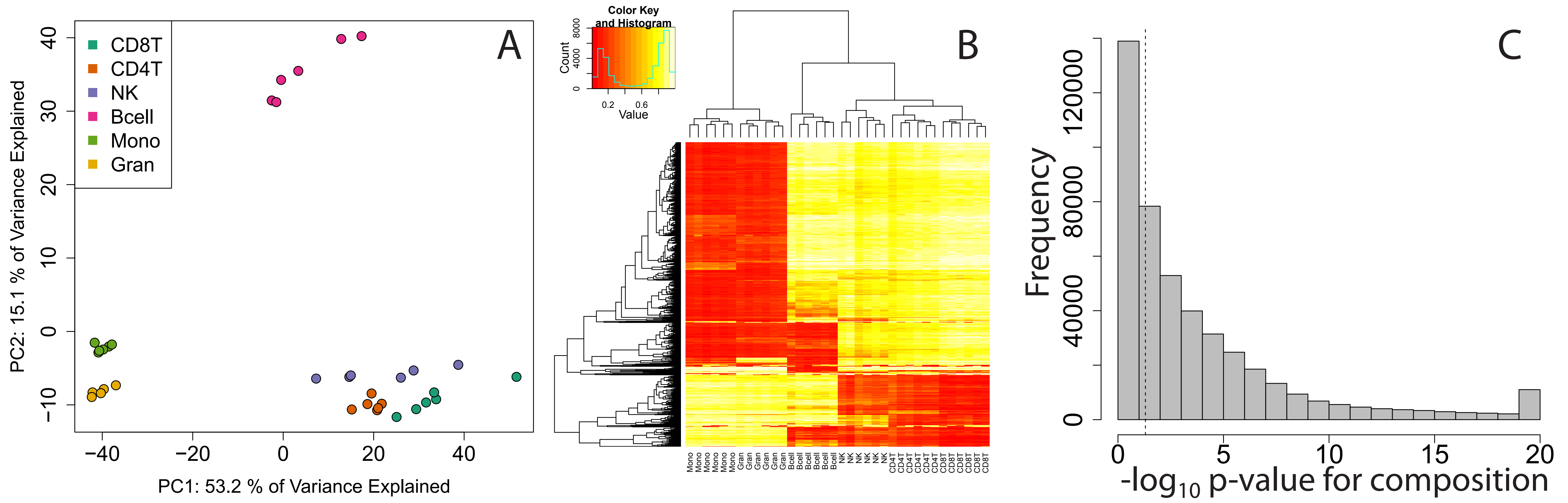

A
CD8T
CD4T
NK
Bcell
Mono
Gran
PC2: 15.1 % of Variance Explained
PC1: 53.2 % of Variance Explained
B
Color Key and Histogram
Count
Value
C
Frequency
-log10 p-value for composition

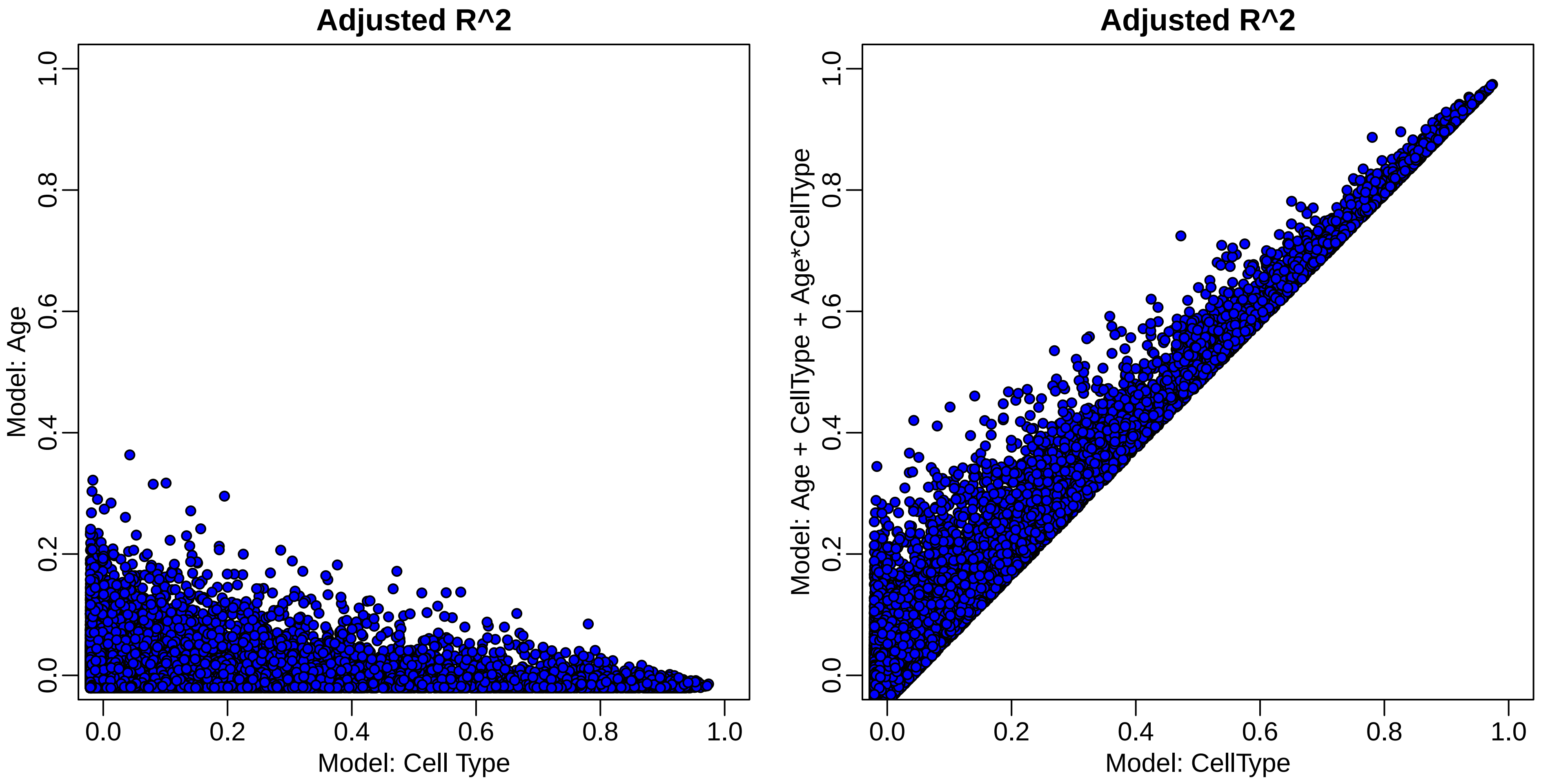

Adjusted R^2
Model: Age
Model: Cell Type
0.0
0.2
0.4
0.6
0.8
1.0
Adjusted R^2
Model: Age + CellType + Age*CellType
Model: CellType

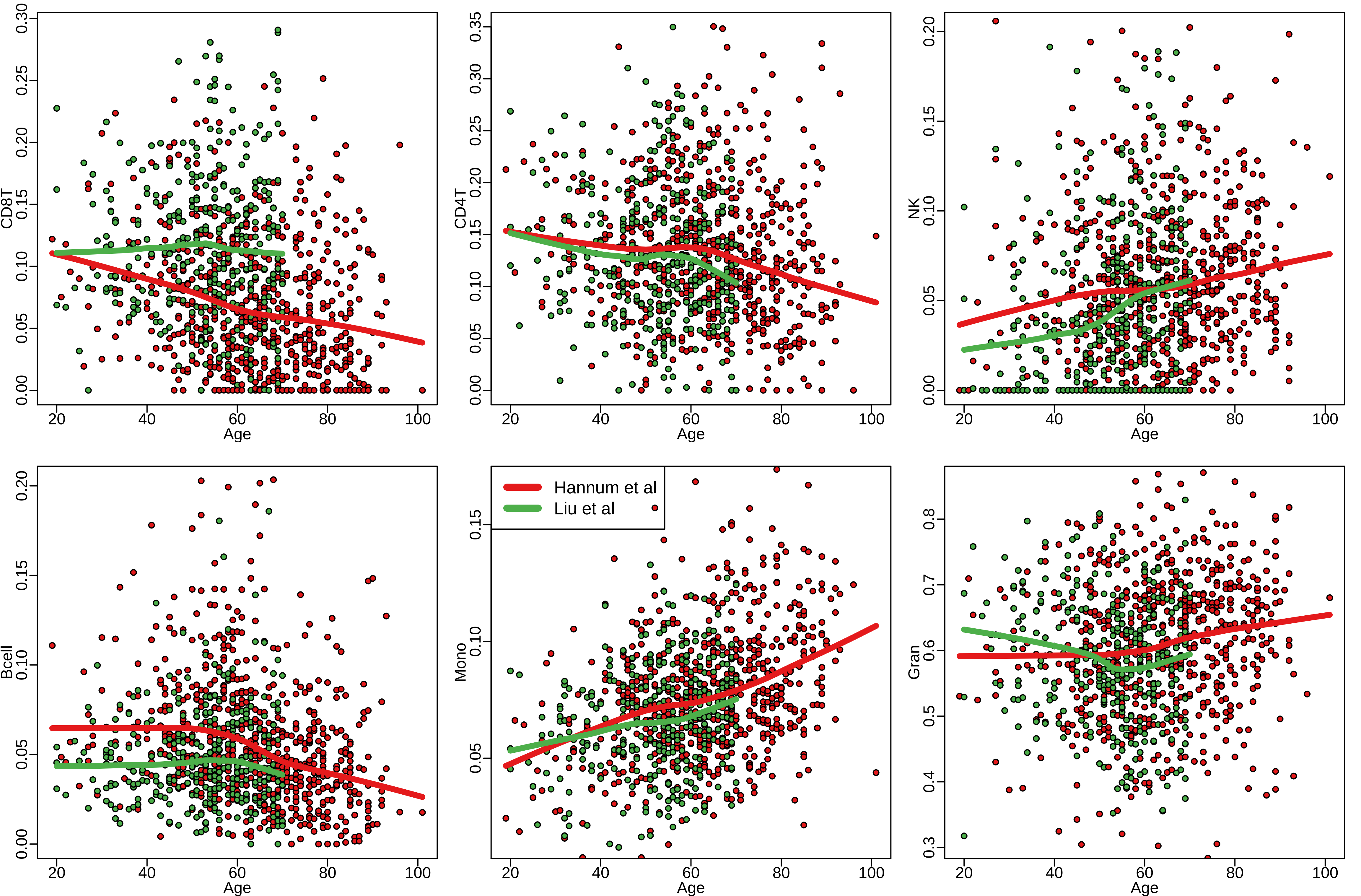

CD8T
CD4T
NK
Bcell
Mono
Gran
Age
Hannum et al
Liu et al

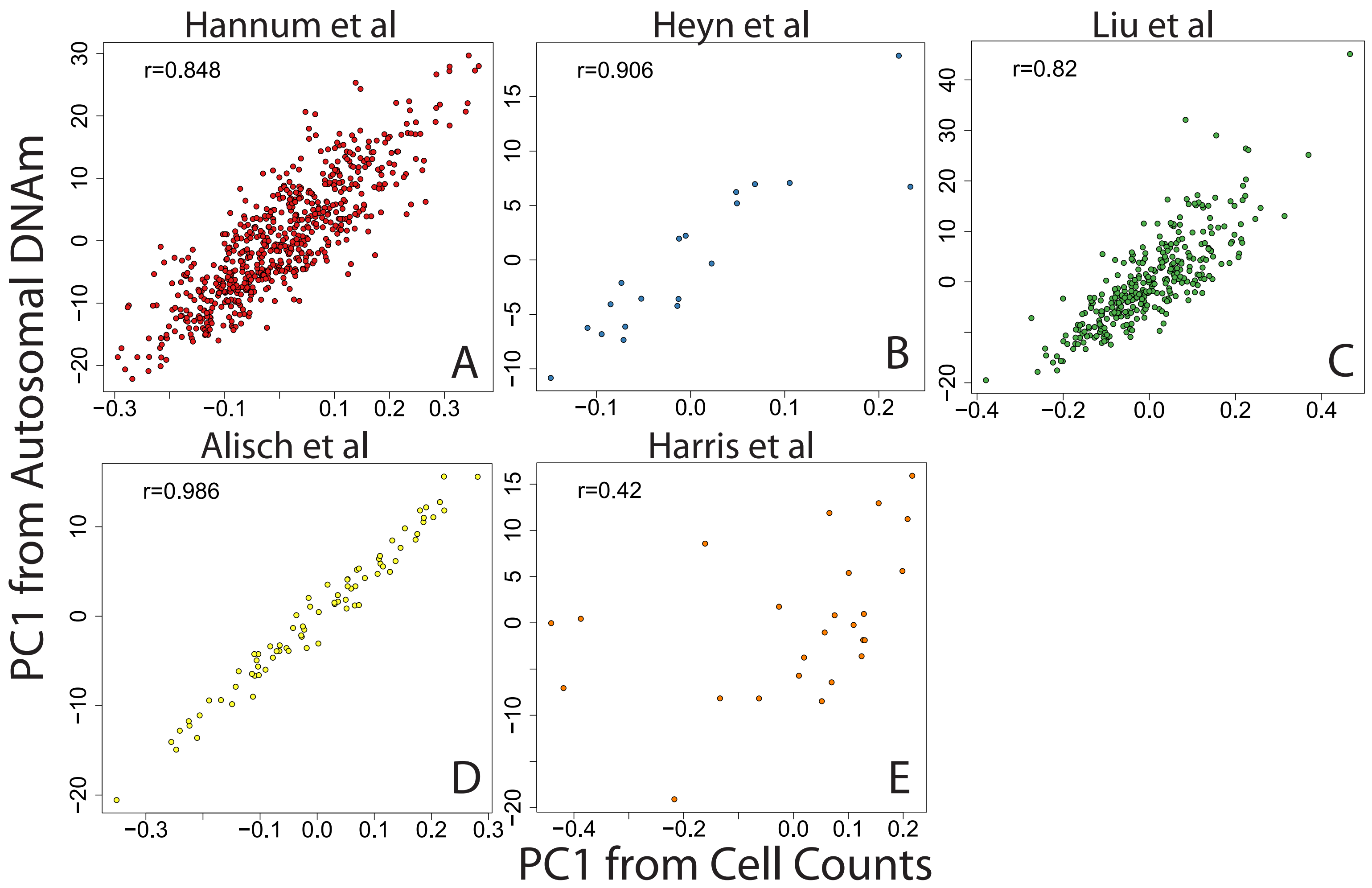

Hannum et al
r=0.848
A
Heyn et al
r=0.906
B
Liu et al
r=0.82
C
Alisch et al
r=0.986
D
Harris et al
r=0.42
E
PC1 from Autosomal DNAm
PC1 from Cell Counts

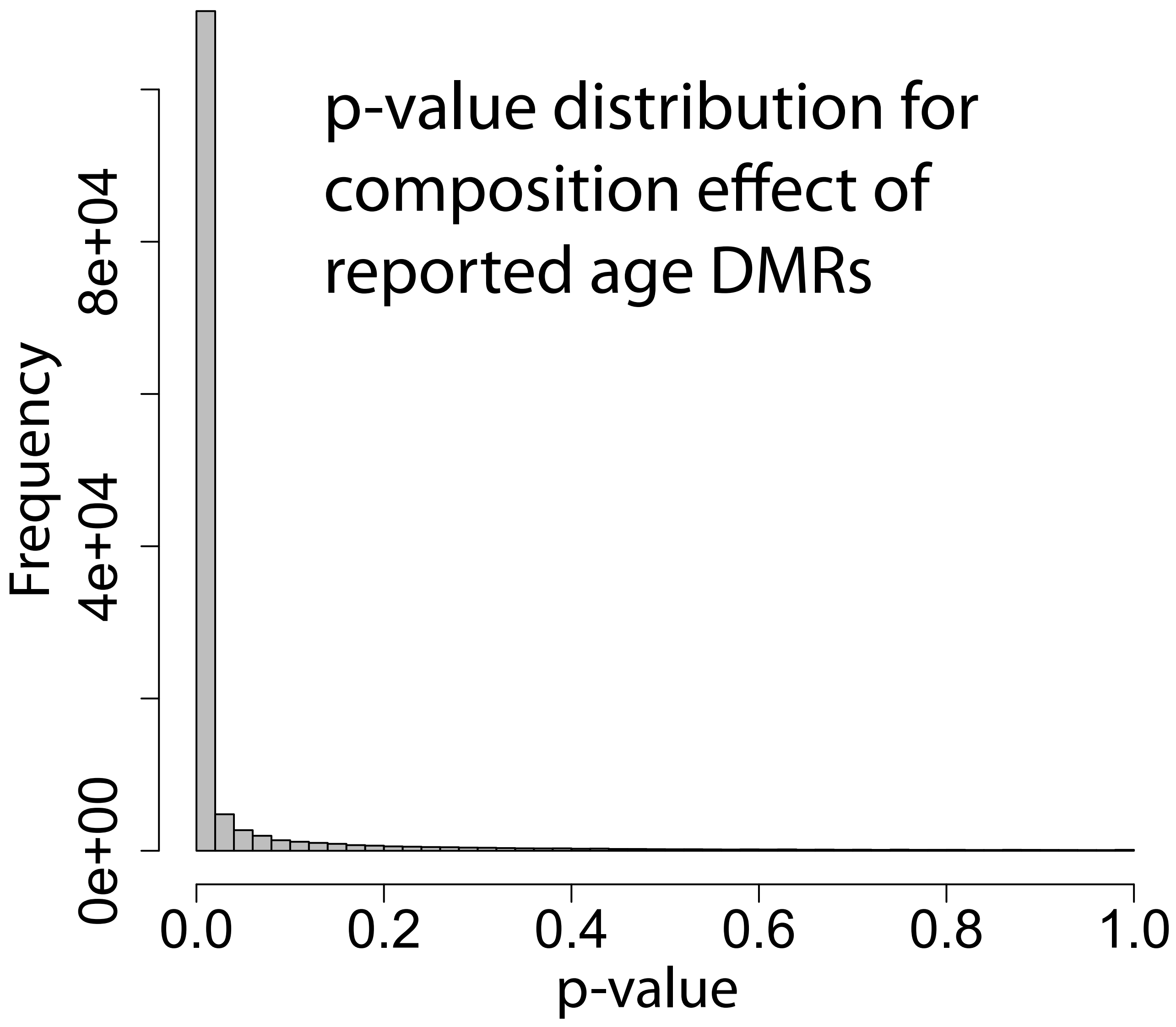
p-value distribution for
composition effect of
reported age DMRs
Frequency
0e+00
4e+04
8e+04
0.0
0.2
0.4
0.6
0.8
1.0
p-value

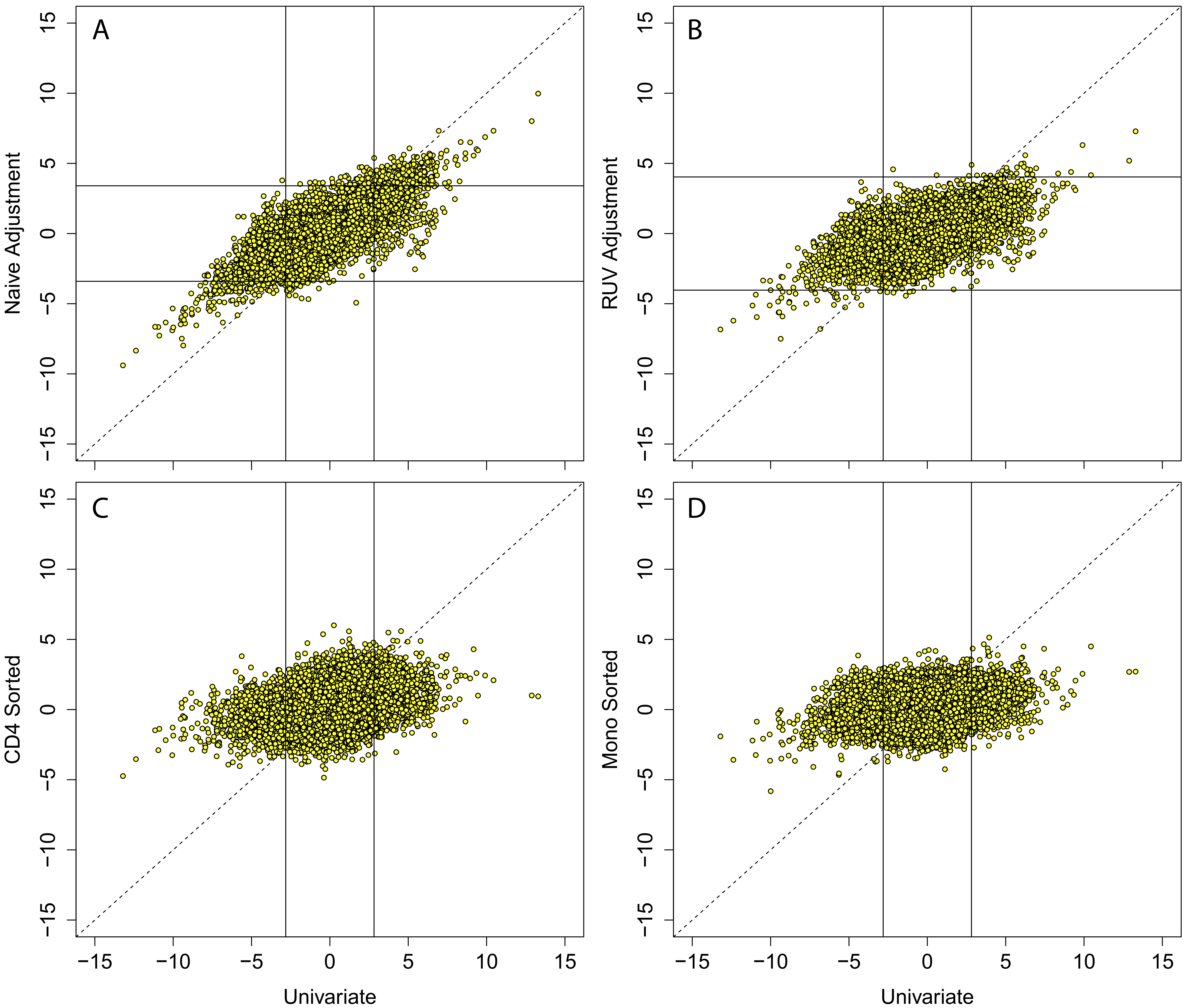

A
B
C
D
Naive Adjustment
RUV Adjustment
CD4 Sorted
Mono Sorted
Univariate
Univariate
−15
−10
−5
0
5
10
15

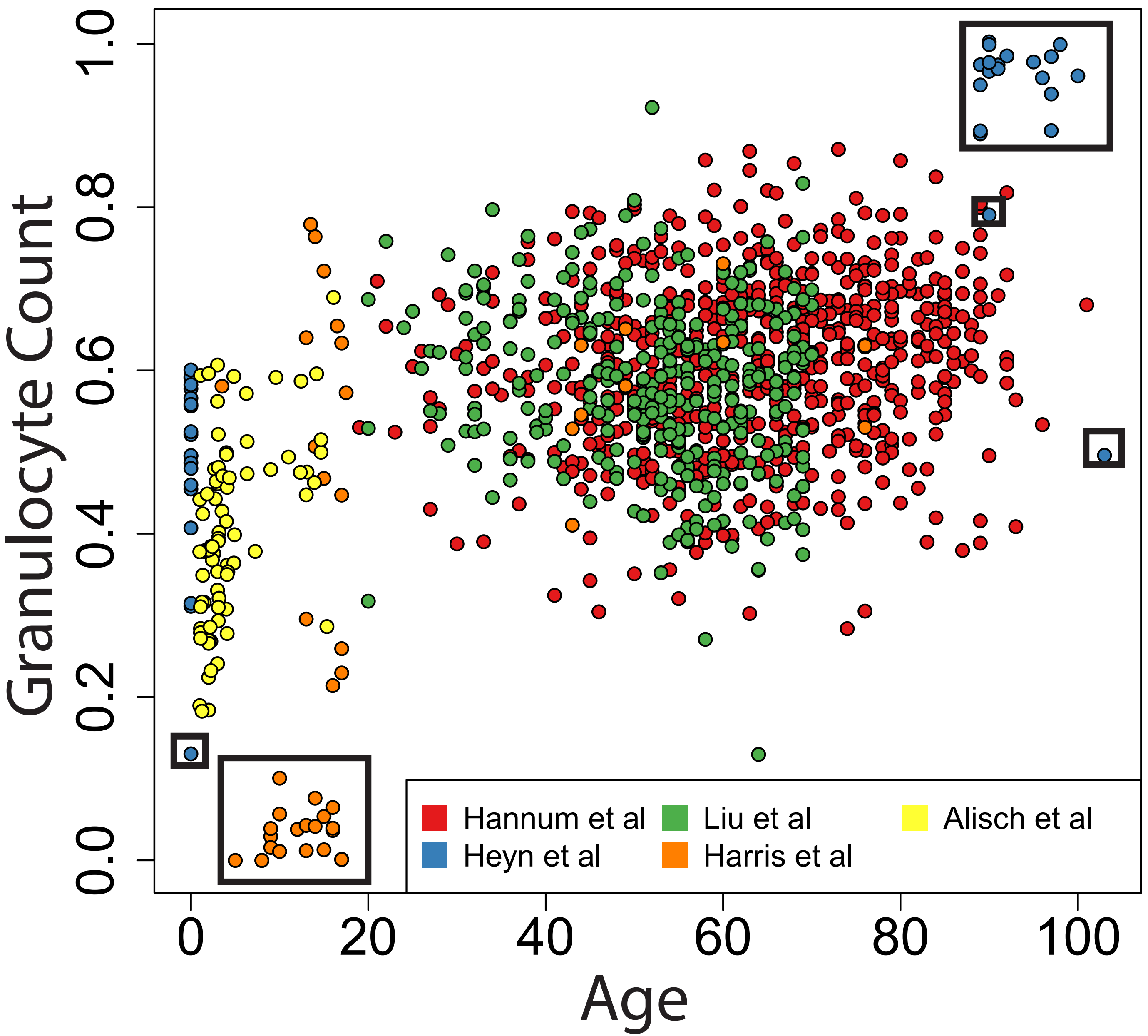
Granulocyte Count
1.0
0.8
0.6
0.4
0.2
0.0
0
20
40
60
80
100
Age
Hannum et al
Liu et al
Alisch et al
Heyn et al
Harris et al

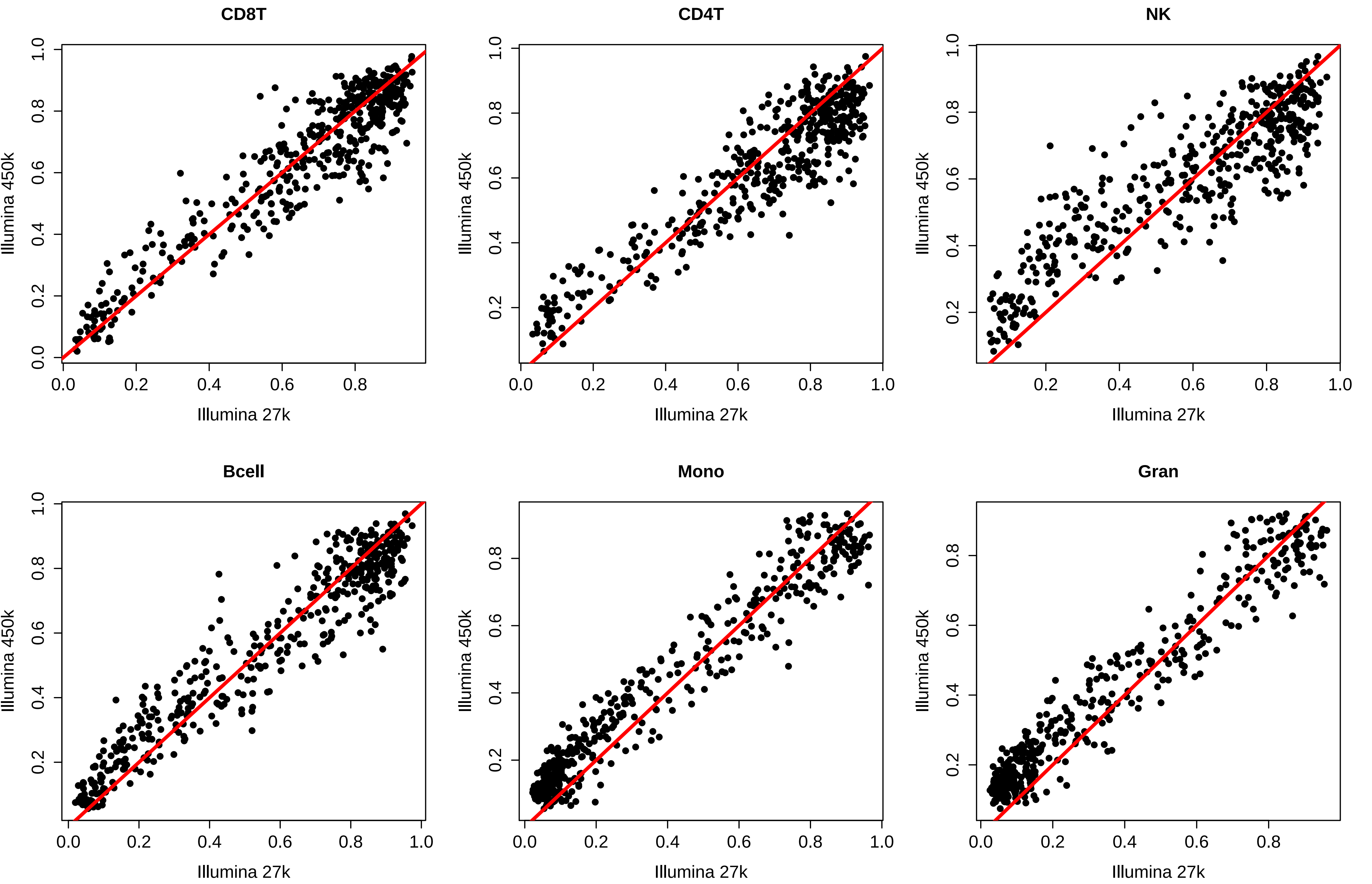

CD8T
CD4T
NK
Bcell
Mono
Gran
Illumina 450k
Illumina 27k
0.0
0.2
0.4
0.6
0.8
1.0

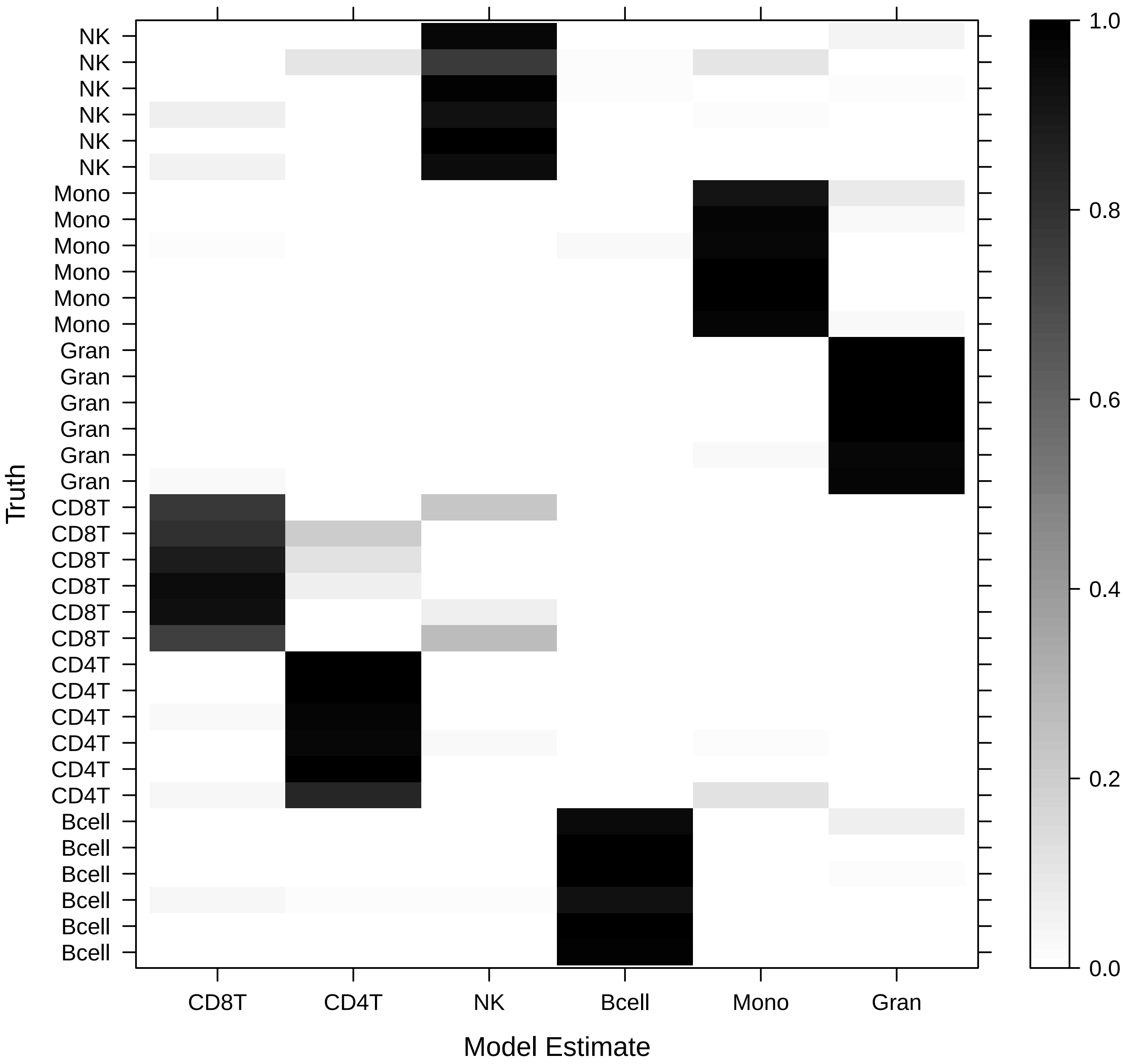

NK
NK
NK
NK
NK
NK
Mono
Mono
Mono
Mono
Mono
Mono
Gran
Gran
Gran
Gran
Gran
Gran
CD8T
CD8T
CD8T
CD8T
CD8T
CD8T
CD4T
CD4T
CD4T
CD4T
CD4T
CD4T
Bcell
Bcell
Bcell
Bcell
Bcell
Bcell
Truth
CD8T
CD4T
NK
Bcell
Mono
Gran
Model Estimate
1.0
0.8
0.6
0.4
0.2
0.0

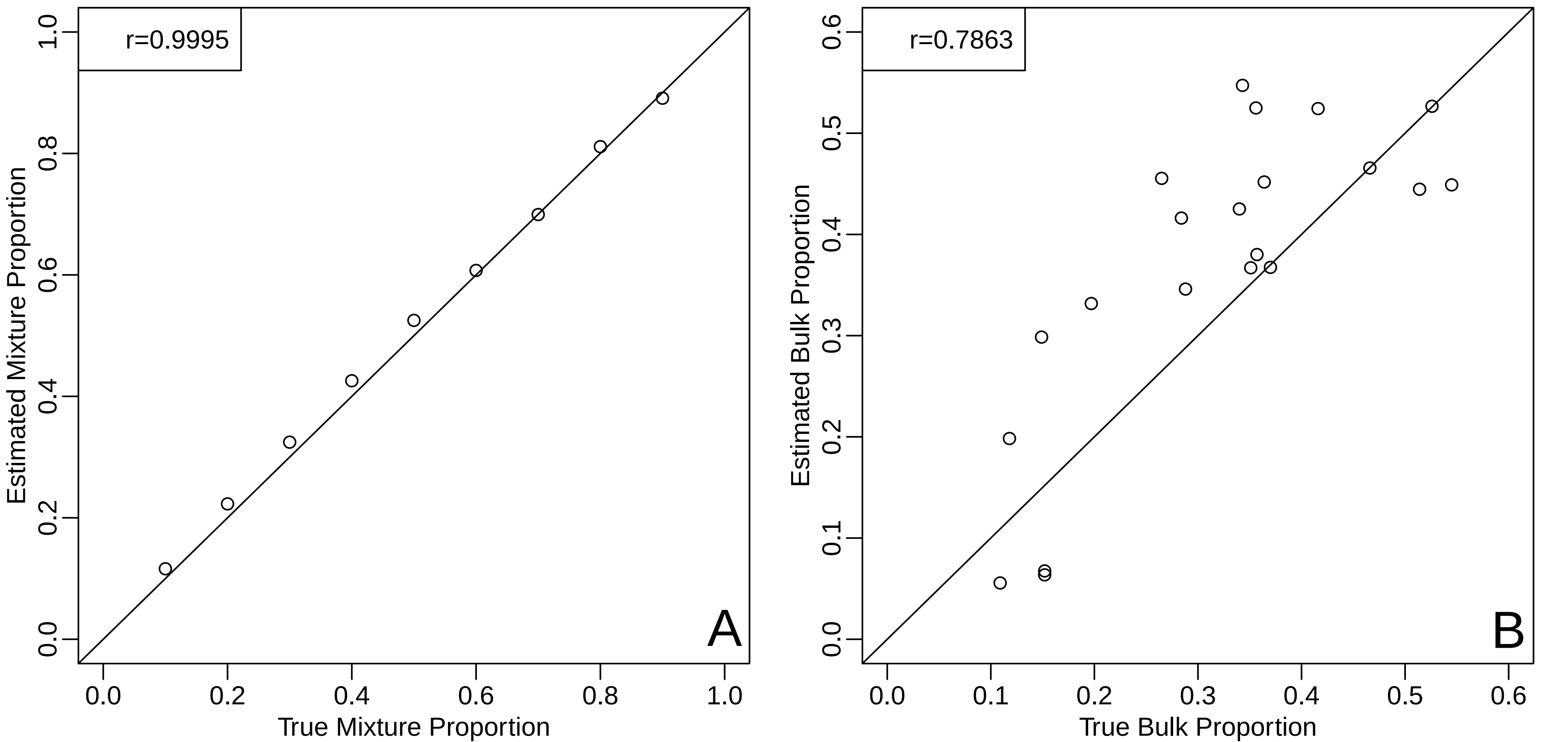
r=0.9995
Estimated Mixture Proportion
True Mixture Proportion
0.0
0.2
0.4
0.6
0.8
1.0
A
r=0.7863
Estimated Bulk Proportion
True Bulk Proportion
0.0
0.1
0.2
0.3
0.4
0.5
0.6
B
